# Promising Colloidal Rhenium Disulfide Nanosheets: Preparation and Applications for In Vivo Breast Cancer Therapy

**DOI:** 10.3390/nano12111937

**Published:** 2022-06-06

**Authors:** Yiwan Song, Yufeng Yuan, Xiao Peng, Zheng Peng, Hao Liu, Yingxin Zhou, Xiaoying Zhang, Feifan Zhou, Jun Song, Junle Qu

**Affiliations:** 1 College of Physics and Optoelectronic Engineering, Shenzhen Key Laboratory of Photonics and Biophotonics, Key Laboratory of Optoelectronic Devices and Systems of Ministry of Education and Guangdong Province, Shenzhen University, Shenzhen 518060, China; yiwansong_syw@163.com (Y.S.); zheng_peng1995@163.com (Z.P.); 20222022@163.com (H.L.); yls772@163.com (Y.Z.); ffzhou@szu.edu.cn (F.Z.); songjun@szu.edu.cn (J.S.); jlqu@szu.edu.cn (J.Q.); 2 School of Electronic Engineering and Intelligentization, Dongguan University of Technology, Dongguan 523808, China; 3 Department of Pharmacology, College of Pharmacy, Shenzhen Technology University, Shenzhen 518118, China; zhangxiaoying@sztu.edu.cn

**Keywords:** PEG-ReS_2_ nanosheets, photothermal conversion efficiency, in vivo photothermal therapy, therapeutic outcomes, miRNA expression analysis

## Abstract

Photothermal therapy (PTT) has become an important therapeutic strategy in the treatment of cancer. However, exploring novel photothermal nanomaterials with satisfactory biocompatibility, high photothermal conversion efficiency, and efficient theranostic outcomes, remains a major challenge for satisfying clinical application. In this study, poly-ethylene glycol modified rhenium disulfide (PEG-ReS_2_) nanosheets are constructed by a simple-liquid phase exfoliation method. The PEG-ReS_2_ nanosheets were demonstrated to have good solubility, good biocompatibility, low toxicity, and strong capability of accumulating near-infrared (NIR) photons. Under 808 nm laser irradiation, the PEG-ReS_2_ nanosheets were found to have an excellent photothermal conversion efficiency (PTCE) of 42%. Moreover, the PEG-ReS_2_ nanosheets were demonstrated to be ideal photothermal transduction agents (PTAs), which promoted rapid cancer cell death in vitro and efficiently ablated tumors in vivo. Interestingly, the potential utility of up-regulation or down-regulation of miRNAs was proposed to evaluate the therapeutic outcomes of PEG-ReS_2_ nanosheets. The expression levels of a set of miRNAs in tumor-bearing mice were restored to normal levels after PTT therapy with PEG-ReS_2_ nanosheets. Both down-regulation miRNAs (miR-125a-5p, miR-34a-5p, miR-132-3p, and miR-148b-3p) and up-regulation miRNAs (miR-133a-3p, miR-200c-5p, miR-9-3p, and miR-150-3p) were suggested to be important clinical biomarkers for evaluating therapeutic outcomes of breast cancer-related PTT. This work highlights the great significance of PEG-ReS_2_ nanosheets as therapeutic nanoagents for cancer therapy.

## 1. Introduction

Breast cancer (BC) is the most common malignant tumor in females worldwide. In 2021, global cancer statistics showed that BC had become the highest morbidity of cancer type. [1] Although BC is the most serious cancer among women, it can be treatable when diagnosed and treated at an early stage. Early stage diagnosis and the choice of specific therapy for BC are crucial, because they can significantly increase patient survival rate and life quality. At present, standard approaches for the treatment of BC include surgery, chemotherapy, radiotherapy [2], and targeted therapy [3]. However, these conventional treatments are still unsatisfactory, due to tumor heterogeneity, severe side effects, time-consuming procedures, and slow treatment outcomes [4]. Occasionally, both chemotherapy and radiotherapy methods fail to treat BC due to severe side effects.

To address these limitations in conventional methods, the development of novel, precise, efficient, and less toxic cancer therapeutic strategies is critical. Breakthroughs in nanotechnology have led to the real possibility of developing novel cancer treatments. In particular, cancer nanotechnology facilitated by functional nanomaterials has been shown to be a useful tool for performing early-stage cancer detection, diagnosis, and treatment. Due to their intriguing photothermal features, a host of less toxic nanomaterials such as zero-dimensional materials (e.g., quantum dots [5,6]), one-dimensional materials (e.g., nanotubes [7]), and two-dimensional (2D) materials (e.g., nanosheets [8]) have been successfully employed in BC diagnosis and therapy. In terms of emerging cancer therapies, the four main treatment approaches include immunotherapy [9] gene therapy [10] photodynamic therapy (PDT) [11] and photothermal therapy (PTT) [12]. These therapeutic strategies can significantly enhance therapeutic outcomes.

Near-infrared (NIR) laser is an ideal light source for performing PTT, because the NIR region is a transparency window for biological tissues [13] Under the illumination of a NIR laser, functional nanomaterials are considered to be excellent photothermal transduction agents (PTAs), which can efficiently harvest NIR photons and produce heat to induce localized hyperthermia. PTT mainly utilizes the photothermal effect of PTAs to kill cancer cells and ablate tumors [14,15,16]. Compared with other therapeutic strategies, PTT has several advantages, including non-invasiveness, low side effects, and high efficiency [17,18,19,20,21]. In addition, it is easy to precisely control the laser irradiation dosage on the tumor to guarantee that any collateral damage to normal tissues can be avoided. Furthermore, PTT has been widely employed to ablate various types of cancers. Excellent PTAs should have certain properties, including high photothermal conversion efficiency (PTCE), high capability of absorbing NIR photons, and good accumulation in tumors. In particular, nanoscale PTAs are ideal to leverage on the enhanced permeability and retention effect, which can help PTAs accumulate in tumors [19]. In addition, organic small molecules have smaller absorbance cross section than that of nanoscale materials. Thus, nanoscale PTAs usually have higher PTCE than small molecular PTAs [22]. Moreover, the intriguing optical features indicated that it is possible to build an integrated nanoplatform including bioimaging modes and therapeutic functions involved by nanoscale PTAs. 

Among a host of PTAs, nanoscale 2D materials are promising nanoplatforms that can be used for diagnosing and treating cancer. Notably, 2D nanomaterials usually have high specific surface areas, which can transport large amounts of drugs into tumors via various pathways. Functional graphene nanomaterial was the first member of the 2D materials family, and has been demonstrated to have a practical application in cancer studies [23,24]. With an increasing number of new members joining the 2D materials family [25,26,27,28], the focus has turned to developing cancer therapeutic strategies based on novel 2D materials. Transition metal dichalcogenides (TMDs) are a family of graphene analogues, comprised more than 40 members [29], which include MoS_2_ [8], WS_2_ [26], and MoSe_2_ [30]. TMD nanosheets show great promise in tumor PTT, due to their high PTCEs and strong NIR absorption. For example, 1T phase MoS_2_ was demonstrated to have a thermal conversion of 43.3% [31], while MoSe_2_ had a photothermal conversion of 57.9% [32]. 

Unlike other members of the TMD family such as MoS_2_ and WS_2_, a new member rhenium disulfide (ReS_2_) is unusual, because its crystal structure has significant anisotropy. Moreover, functional ReS_2_ nanosheets have great potential for biomedical studies due to their strong NIR absorption and high PTCE. Generally, functional ReS_2_ nanosheets can be prepared by both bottom-up and top-down methods. For example, using the bottom-up method, Shen et al. [33] prepared uniform ReS_2_ nanosheets that were employed for cancer photothermal radiotherapy, while Miao et al. [34] constructed colloidal ReS_2_ nanosheets to guide cancer photoacoustic/CT imaging and PTT using a liquid-phase exfoliation approach. Although both cell and animal experimental data have demonstrated the low toxicity of 2D ReS_2_ nanosheets, their potential toxicity in humans remains unclear. Hematoxylin and eosin (H&E) staining of tumor and five major organ slices, including the heart, liver, spleen, lung, and kidney, can be employed to determine the therapeutic outcomes and materials’ toxicity [19,35]. The evaluation of H&E stained tissue sections is a standard in clinical diagnosis, but limited in quantitative analysis. Thus, it is critical to develop a novel method to evaluate the cancer therapy efficiency of functional 2D materials. Circulating microRNAs (miRNAs) are important blood-based cancer biomarkers that could be used to evaluate cancer therapy efficiency [36,37]. Generally, miRNAs are potential oncogenes or tumor suppressors, and changes in miRNA expression are highly correlated with the emergence of cancer. Thus, miRNAs have been considered to be an important molecular tool for performing non-invasive cancer prognosis. By observing changes in the expression of blood circulating miRNAs before and after PTT, the therapeutic outcomes can be determined. To the best of our knowledge, no studies have reported the use of miRNA expression to characterize the therapeutic outcomes of the nanoagent-based PTT.

**Figure 1:**
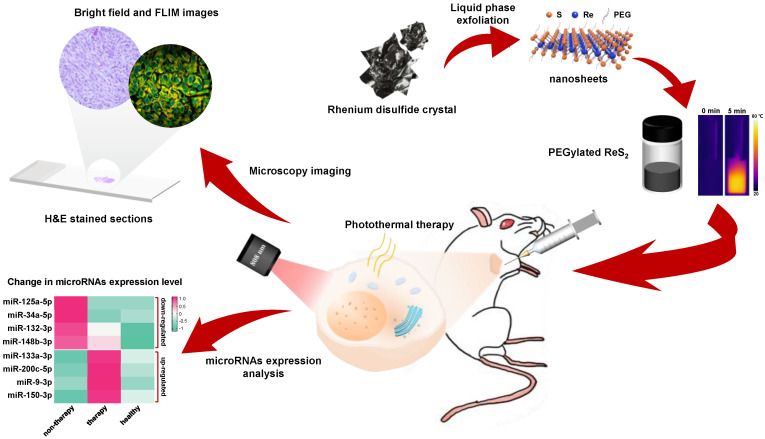
Schematic illustration of PEG−ReS_2_ nanosheets employed for in vivo breast cancer therapy study.

Herein, novel 2D ReS_2_ nanosheets were prepared, and their use in BC therapy was systematically explored. As shown in Figure 1, colloidal ReS_2_ nanosheets were synthesized through a liquid phase exfoliation approach. The addition of a poly-ethylene glycol (PEG) surface modification resulted in functional ReS_2_ nanosheets, which proved to be a promising PTA due to its good solubility and biocompatibility. More importantly, we demonstrated that these functional ReS_2_ nanosheets could be used for in vivo breast cancer therapy, due to their strong NIR light absorption ability at 808 nm and high PTCE of 42%. In addition to H&E-stained tissue sections, we used miRNA expression analysis to assess the PTT outcome from functional ReS_2_ nanosheets. Both up-regulation and down- regulation miRNAs were found to be important indicators for verifying the satisfactory therapeutic effects of PEG-ReS_2_ nanosheets. In summary, our work presents a novel strategy to design high PTCE nanoagents, treat cancer, and characterize PTT outcomes, which may provide a link between animal experiments and clinical studies. 

## 2. Materials and Methods

### 2.1. Chemicals and Materials

The chemicals used in this study were not purified. Solutions were prepared using deionized water with a resistivity of 18.2 MΩ/cm. N-Methyl-2-pyrrolidone (NMP, 99%, Reagent Plus) was purchased from Sigma-Aldrich (Shanghai, China). Rhenium disulfide (ReS_2_) crystals were obtained from HQ Graphene Company (Groningen, The Netherlands). PEG-NH_2_ (MW: 2000 kDa) was purchased from Shanghai Ponsure Biotech, Inc (Shanghai, China). Cell Counting Kit-8 (CCK8) assay kits, calcein acetoxymethyl ester (AM), and propidium iodide (PI) were purchased from KeyGen BioTech (Nanjing, China). 4T1 BC cells were obtained from American Type Culture Collection (ATCC, Manassas, VA, USA). Cell culture reagents such as Dulbecco’s Modified Eagle Medium (DMEM) were purchased from Thermo Fisher SCIENTIFIC (Waltham, MA, USA). 

### 2.2. Exfoliation and Surface Modification of ReS_2_ Nanosheets

ReS_2_ nanosheets were prepared in solvent NMP via a simple liquid-phase exfoliation approach. Bulk ReS_2_ crystals (80 mg) were added to 80 mL NMP and continuously sonicated for 10 h in an ice bath. The ultrasonic exfoliation conditions were as follows: the operation power of the ultrasonic homogenizer was 1200 W, and the lasting time of each ultrasound cycle was 2 s, with a 4 s break between each cycle. To remove large-sized ReS_2_ nanosheets, the mixture containing NMP and ReS_2_ nanosheets was centrifuged at 3000 rpm for 20 min. To obtain smaller sized ReS_2_ nanosheets, the supernatant was centrifuged at 11,000 rpm for 30 min. Finally, the prepared ReS_2_ nanosheets were washed twice with ethanol and ultrapure water, respectively. 

PEGs are a class of biocompatible polymers, which can help nanoscale PTAs target tumor. It is well known that the surface charge of cell membrane is confirmed to be negative [38]. However, the surface charge of nanoscale PTAs functionalized by the –NH_2_ moiety is usually positive. Thus, the electrostatic adsorption interaction can significantly develop uptake the behavior of cancer cells. Moreover, it has been reported that, PEG-NH_2_-decorated nanoscale PTAs have shown high stability and low cytotoxicity [6,39]. The solubility and biocompatibility of the ReS_2_ nanosheets under physiological conditions were enhanced by modifying the surface of the ReS_2_ nanosheets with PEG-NH_2_. Briefly, 50 mg PEG-NH_2_ was added to 100 mL ReS_2_ nanosheet solution and continuously stirred for 12 h. Then, the PEG-ReS_2_ nanosheets were centrifuged at 11,000 rpm three times to remove excess PEG molecules. After surface modification, the PEG-ReS_2_ nanosheets were re-suspended in deionized water.

### 2.3. Characterization of PEG-ReS_2_ Nanosheets 

Transmission electron microscopy (TEM) imaging of the PEG-ReS_2_ nanosheets was performed using a transmission electron microscope (JEM-1230 CX, JEOL Ltd., Tokyo Japan). In addition, the Vis-NIR absorption spectra of PEG-ReS_2_ nanosheets ranging from 400 nm to 900 nm were acquired using a UV-VIS spectrophotometer (UV-1780, SHIMADZU, Kyoto, Japan). Under the excitation of a 785 nm laser, the Raman spectra of the PEG-ReS_2_ nanosheets and bulk ReS_2_ crystals were measured by a confocal Raman spectrometer (Renishaw, inVia, London, UK). 

### 2.4. Measurement of PTCE

The PTCE of the PEG-ReS_2_ nanosheets was determined by examining their photothermal performances. PEG-ReS_2_ nanosheets were irradiated using a NIR laser at 808 nm (power density, 1 W/cm^2^) and the heating curve of various concentrations (25, 50, 100, 200 and 400 μg/mL) of PEG-ReS_2_ nanosheets was measured every 30 s for 5 min. Deionized water was employed as the control group. The photothermal stability of the PEG-ReS_2_ nanosheets was determined by performing five heating-cooling cycles of PEG-ReS_2_ nanosheet solutions. The laser was turned on and the heating curve of the PEG-ReS_2_ nanosheet solution was monitored for 5 min. Then, the laser was turned off, and the cooling curve of the PEG-ReS_2_ nanosheet solution was recorded every 30 s until the temperature naturally decreased to room temperature. Using the heating-cooling curves versus time data, the PTCE (*η*) of PEG-ReS_2_ nanosheets was determined by Equation (1) [21,40]

(1) $$\eta =\frac{hS\Delta T-Q_{s}}{I\left(1-10^{-A_{808}}\right)}$$

where *h* denotes the heat transfer coefficient, and *S* denotes the radiation surface area of the quartz cell. Thus, the value of *hS* can be determined (Supplementary Materials, Supplementary Equations). Δ*T* is the difference between the saturation temperature and surrounding temperature. *Qs* represents the power absorption of the solvent. Finally, I denotes the power density of the incident laser at 808 nm, and *A*_808_ represents the absorbance of PEG-ReS_2_ colloids at 808 nm.

### 2.5. Cytotoxicity Assay 

4T1 cells (10,000 cells/well in a 96-well plate) were cultured in DMEM at 37 °C in an atmosphere of 5% carbon dioxide (CO_2_). A CCK-8 kit was employed to evaluate the biocompatibility of PEG-ReS_2_ nanosheets. After 24-h incubation, PEG-ReS_2_ nanosheets (25, 50, 100, and 200 μg/mL) were added to the cultured 4T1 cells. For each concentration of PEG-ReS_2_ nanosheets, six replicates were performed. After incubating for a further 24 h, the mixture solutions were removed, and the cells were incubated with 10 μL CCK-8 and 100 μL culture medium for 1 h. The optical density (OD) value at 450 nm wavelength was obtained using a microplate reader.

### 2.6. In Vitro Photothermal Therapy

4T1 cells were cultured in 96-well plates at 37 °C in an atmosphere of 5% CO_2_ for 24 h. Next, the culture medium was replaced with various PEG-ReS_2_ nanosheet solutions (0, 50, 100, and 200 μg/mL). For each concentration of PEG-ReS_2_ nanosheets, four replicates were performed. After 1 h of incubation with the PEG-ReS_2_ nanosheets, the 4T1 cells were irradiated for 5 min using an 808 nm laser (1 W/cm^2^). Then, the 4T1 cells were incubated with CCK-8 solution for one hour. The cell viability of the 4T1 cells was measured by microplate reader. 

To visually evaluate the PTT outcomes of PEG-ReS_2_ nanosheets, the live/dead cell double staining kit was employed. After the 808 nm laser irradiation for 10 min, live/dead 4T1 cells were incubated for 30 min by both calcein acetoxymethyl ester (2 µM) and propidium iodide (8 µM) solutions. Then, the cells samples were imaged by a Nikon A1R MP system. 

### 2.7. Animal Experiments

Animal experiments were performed in accordance with the guidelines of the Animal Ethical and Welfare Committee of Shenzhen University (AEWC-SZU). Healthy BALB/c mice were purchased from Beijing Vital River Laboratory Animal Technology Co., Ltd. (Beijing, China). Thirty-six healthy female mice (approximately 20 g) were selected for the in vivo PTT experiments. Previous studies [27,41] have reported subcutaneous injection of 4T1 cells into the hind limbs of mice to study in vivo PTT on BC cells. However, this method does not allow the therapeutic outcomes of BC to be properly evaluated. Thus, we constructed a reliable breast tumor model, by subcutaneously injecting approximately 5 × 10^5^ of 4T1 cells into the left breast pad of the mice. Afterwards, both the tumor size and weight of each mouse were recorded during the tumor growth process. 

### 2.8. In Vivo Photothermal Therapy

When the tumor volume of the mice reached 80 mm^3^, the mice were randomly divided into four groups (nine mice in each group): (Group 1) PBS; (Group 2) NIR; (Group 3) ReS_2_; (Group 4) ReS_2_ + NIR. The tumors of both Group 1 and Group 2 mice were injected with 100 μL PBS, while the tumors of Group 3 and Group 4 mice were injected with 100 μL PEG-ReS_2_ nanosheet solution (2 mg/mL). After 1 h, the mice in Group 2 and Group 4 were anesthetized, and their tumors were irradiated using a NIR 808 nm laser (0.5 W/cm^2^) for 10 min. During PTT, an infrared thermography was employed to record the temperature of the tumor surface. On the second day, one mouse from each group was randomly selected, sacrificed and tumor slices were prepared. The tumor volume was measured every 2 days for a total of 30 days in the remaining mice. On day 14, mice in Group 1, Group 2 and Group 3 presented with large tumor volumes and had to be sacrificed. Both the tumor and vital organs including the heart, liver, spleen, lung, and kidney were collected, and tissue slices were prepared and stained with H&E. On day 30, the same procedure was performed on Group 4 mice.

### 2.9. In Vivo Toxicity Analysis

To perform the in vivo toxicity analysis of PEG-ReS_2_ nanosheets, two healthy BALB/c mice were injected with 100 μL PEG-ReS_2_ (6 mg/mL in water) via the tail vein. Two healthy mice in the Control group were injected with 100 μL PBS solution via the tail vein. Two months post-injection, the five major organs, including heart, liver, spleen, lung, and kidney were collected and H&E-stained tissue sections were prepared.

### 2.10. Optical Imaging of Tissue-Stained Sections

H&E-stained tissue slices including tumors and organs were examined using a Nikon microscope equipped with a 20× objective lens. In addition, fluorescence lifetime images were obtained using a fluorescence lifetime imaging microscopy (FLIM) system with a 100× objective lens. The TE-2000U (Nikon, Tokyo, Japan) microscope was equipped with a time-correlation single photon counting module DCS120 (Becker & Hickl GmbH, Berlin, Germany). 

### 2.11. miRNA Expression Profiling

To perform miRNA expression analysis, three healthy BALB/c mice were selected as blank controls. In addition, three mice were selected from Group 1 (PBS) and Group 4 (ReS_2_ + NIR), and divided into non-therapy and therapy groups, respectively. On day 14 post-treatment, blood samples were collected via submandibular bleeding. Then, serum was extracted by centrifuging the blood samples at 3000× *g* for 5 min. 

The serum samples were mixed with Trizol (Thermo Fisher Scientific, Waltham, MA, USA), and total RNA was extracted using a Direct-zolTM RNA MiniPrep kit (ZYMO Research). Clean sequencing reads were obtained using deep sequencing technology (Huada Gene, Shenzhen, China). In addition, Bowtie was used to map clean reads to the reference genome and to other sRNA databases [41]. Next, miRDeep2 was employed to predict novel miRNAs by exploring the characteristic hairpin structure of the miRNA precursor [42]. Small RNA expression levels were calculated using Transcripts Per Kilobase Million (TPM) [43]. The TPM method eliminates the influence of sequencing discrepancy on the calculation of small RNA expression levels. Differentially expressed gene (DEGs) sequences were counted based on a Poisson distribution using previously reported strategies to correct *p*-values to Q-values [44]. In order to improve the accuracy of DEGs, we defined genes with fold change > 2 and Q-value ≤ 0.001 as significant DEGs [45]. 

## 3. Results and Discussion

**Figure 2:**
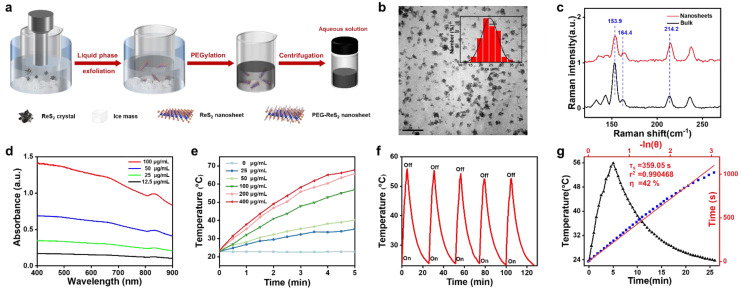
(**a**) Preparation strategy of PEG-ReS_2_ nanosheets. (**b**) TEM image of PEG-ReS_2_ nanosheets. (**c**) Raman spectrum of bulk ReS_2_ and PEG-ReS_2_ nanosheets. (**d**) Absorbance spectra of PEG-ReS_2_ nanosheets at various concentrations (12.5, 25, 50, 100 μg/mL). (**e**) Photothermal heating curves of PEG−ReS_2_ nanosheets in water at various concentrations (0, 25, 50, 100, 200, 400 μg/mL). The irradiation laser was 808 nm, with a power density of 1 W/cm^2^ and an irradiation time of 5 min. (**f**) Photothermal heating and cooling curves of 100 μg/mL PEG-ReS_2_ nanosheets in water were obtained from five laser on/off cycles. The irradiation laser was 808 nm, with a power density of 1 W/cm^2^. Within each heating-cooling cycle, the laser was turned on for 5 min. When the laser was turned off, the temperature of the PEG-ReS_2_ nanosheets in water cooled down to room temperature before the laser was turned on again. (**g**) Plots of linear fitting cooling time versus negative natural logarithm of driving force temperature based on the heating and cooling curves in Figure 2f. The time constant (τ_s_) of heat transfer is 359.05, and the PCE of PEG-ReSe_2_ nanosheets can be calculated to be 42%.

As a new member of the TMD family, ReS_2_ crystals are a van der Waals (vdW) layered material, with weak vdW interactions between the stacking layers, making it possible to exfoliate 2D ReS_2_ nanosheets via external force. Figure 2a shows the liquid-phase exfoliation strategy used for preparing 2D ReS_2_ nanosheets. The probe sonication approach and ice-bath conditions were used to exfoliate 2D ReS_2_ nanosheets from bulk ReS_2_ crystals in liquid NMP. PEG was used to coat the surface of ReS_2_ nanosheets to improve their solubility and biocompatibility. With the help of this surface modification, the PEG-ReS_2_ nanosheets showed good dispersibility. The PEG-ReS_2_ nanosheets were further characterized by TEM and Raman spectroscopy (Figure 2b,c). The average size of the PEG-ReS_2_ nanosheets was 20–30 nm, which would allow entry into living cells via the endocytosis method [46,47]. In addition, Raman spectroscopy was employed to determine the chemical composition of the PEG-ReS_2_ nanosheets. Two Raman peaks located at 153.9 cm^−1^ and 164.4 cm^−1^ were attributed to the in-plane vibrational mode, and a Raman band at 214.2 cm^−1^ was attributed to the out-of-plane vibrational mode [48,49]. Unlike other TMDs, PEG-ReS_2_ nanosheets showed strong absorption in the NIR region (Figure 2d). A typical band located at 834 nm was observed, indicating that PEG-ReS_2_ nanosheets had significant capability of absorbing NIR photons.

To further examine the photothermal properties of PEG-ReS_2_ nanosheets, three important factors (PTCE, photothermal stability, and biocompatibility) were systemically studied. Various concentrations (25, 50, 100, 200, and 400 μg/mL) of PEG-ReS_2_ nanosheets in water were exposed to laser irradiation at 808 nm for 5 min, and the photothermal heating curves were measured. As shown in Figure 2e, the PEG-ReS_2_ nanosheets produced a significant photothermal effect. Moreover, the photothermal effect was highly correlated with the concentration of PEG-ReS_2_ nanosheets. For example, in 200 μg/mL PEG-ReS_2_ nanosheets, a temperature increment of 42.2 °C was observed in 5 min, while a temperature increment of only 0.1 °C was observed in deionized water. These findings indicated that PEG-ReS_2_ nanosheets have the ability to rapidly absorb NIR photons and generate efficient thermal energy. In addition, the photothermal stability of PEG-ReS_2_ nanosheets was evaluated by performing five photothermal heating-natural cooling cycles. As shown in Figure 2f, PEG-ReS_2_ nanosheets maintained good photothermal stability after five heating-cooling cycles, indicating that the PEG-ReS_2_ nanosheets have the potential to be used as PTAs. Based on Equation (1), the PTCE of the PEG-ReS_2_ nanosheets was approximately 42% (Figure 2g), which was relatively high compared to other commercial Au nanoshells (13%) [13], Au nanorods (21%) [13], Cu_9_S_5_ Nanocrystals (25.7%) [50], and BP QDs (28.4%) [6].

**Figure 3:**
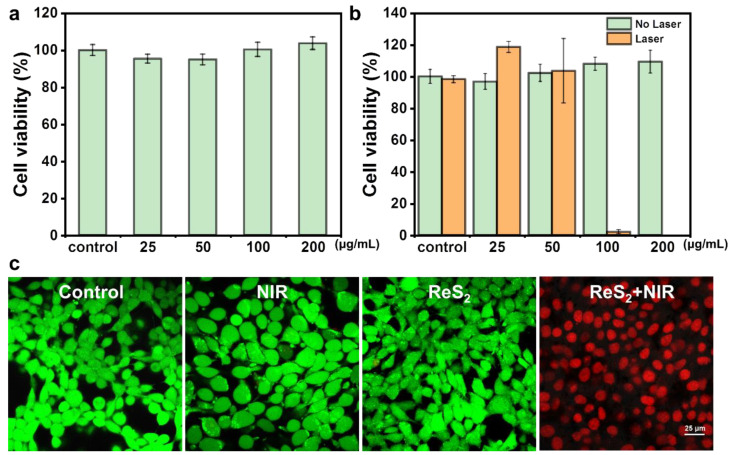
(**a**) Relative 4T1 cell viabilities after incubation with various concentrations of PEG-ReS_2_ nanosheets (0, 25, 50, 100, 200 μg/mL) for 24 h. (**b**) Relative 4T1 cell viabilities after incubation with various concentrations of PEG-ReS_2_ nanosheets (0, 25, 50, 100, 200 μg/mL) and irradiation with an 808 nm laser (1 W/cm^2^) for 5 min. (**c**) Confocal images of treated 4T1 cells (Control, NIR, ReS_2_, and ReS_2_ + NIR) co-stained by calcein AM (green fluorescence) and PI (red fluorescence) solutions.

Nanomaterials that are employed for PTT must have good biocompatibility. Thus, the cytotoxicity of PEG-ReS_2_ nanosheets was evaluated using CCK-8 assays to determine the effects of PEG-ReS_2_ nanosheets on cell viability. No significant cytotoxic effects were observed when various concentrations of PEG-ReS_2_ nanosheets were incubated with 4T1 cells for 24 h in the absence of 808 nm laser irradiation (Figure 3a). Even after incubation with the highest concentration (200 μg/mL) of PEG-ReS_2_ nanosheets, normal 4T1 cell growth was observed, indicating that PEG-ReS_2_ nanosheets have good biocompatibility, which is necessary for biomedical applications. We also examined the in vitro PTT of 4T1 cells by incubating 4T1 cells with various concentrations of PEG-ReS_2_ nanosheets (0, 25, 50, 100, and 200 μg/mL) for 1 h, then irradiating cells with an 808 nm laser (powder density, 1 W/cm^2^) for 5 min. As shown in Figure 3b, significant 4T1 cell death was observed at concentrations of PEG-ReS_2_ nanosheets higher than 100 μg/mL, while exposure to 200 μg/mL PEG-ReS_2_ nanosheets alone was not toxic to 4T1 cells. To visualize the PTT effects from PEG-ReS_2_ nanosheets, treated 4T1 cells were co-stained by both calcein acetoxymethyl ester and propidium iodide solutions. Calcein acetoxymethyl ester can emit green fluorescence in live cells, while propidium iodide is a red fluorescent nucleic acid stain permeating only the damaged cells. Under various treatments (Control, NIR, ReS_2_, and ReS_2_ + NIR), the 4T1 cells were imaged, as shown in Figure 3c. It can be found that there are no dead cells in the three groups (Control, NIR, and ReS_2_), and the 4T1 cells still show high cell viability. However, almost all the 4T1 cells were killed by the PTT effects from PEG-ReS_2_ nanosheets. Thus, our findings suggest that the significant cell death was due to the excellent photothermal effects of the PEG-ReS_2_ nanosheets.

**Figure 4:**
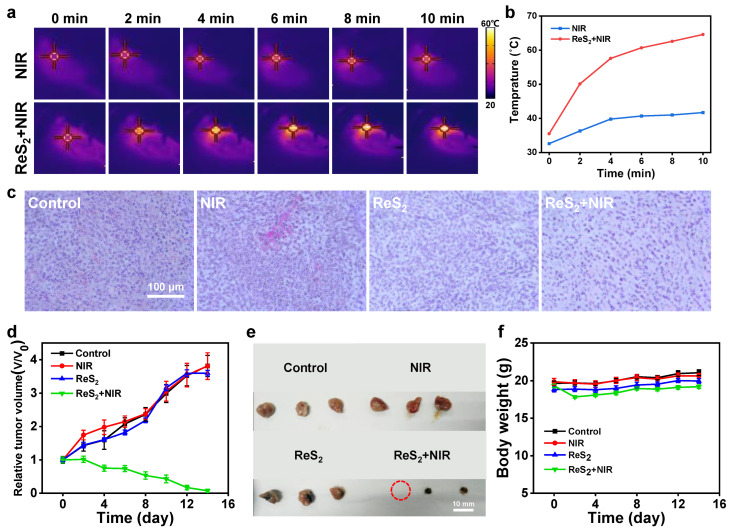
In vivo photothermal therapy. (**a**) Infrared thermal images of tumor-bearing mice injected with PBS and PEG-ReS_2_, respectively. The power density of the 808 nm laser was 0.5 W/cm^2^, and the irradiation time was 10 min. (**b**) The temperature curves at the tumor site according to Figure 4a. (**c**) Representative images of the H&E-stained tumor slices from four experimental groups one day after PTT treatment. (**d**) After PTT, the relative tumor volume of tumor-bearing mice was measured in the four experimental groups (8 mice in each group) every two days. (**e**) At day 14, tumors were obtained from three randomly selected mice from each group for comparison. Tumors in the ReS_2_ + NIR group were ablated, while tumors in the other groups were still large in size. (**f**) The body weight of the tumor-bearing mice from four experimental groups was measured.

Next, we examined the effects of the PEG-ReS_2_ nanosheets in our in vivo PTT model. We constructed an orthotopic breast tumor model by subcutaneously injecting 4T1 cells into the breast pad of BALB/c mice. When the tumors reached a size of approximately 80 mm^3^, the tumor-bearing mice were divided into four groups: (Group 1) Control; (Group 2) NIR; (Group 3) ReS_2_; and (Group 4) ReS_2_ + NIR. Group 1 and Group 2 mice were intratumorally injected with 100 μL PBS solution, while Group 3 and Group 4 mice were intratumorally injected with 100 μL PEG-ReS_2_ (2 mg/mL) nanosheet solution. Group 2 and Group 4 mice were anesthetized and their tumor sites were treated with an 808 nm laser (0.5 W/cm^2^) for 10 min. During the PTT process, the real time temperature at the tumor sites was instantaneously recorded by infrared thermography (Figure 4a). During the 10-min laser treatment, the temperature at the Group 2 tumor sites increased from 32.6 °C to 41.7 °C, an increment of 9.1 °C. However, in Group 4 mice, the real time temperature at the tumor sites increased significantly from 35.5 °C to 64.6 °C, a temperature increment of 29.1 °C (Figure 4b). Previous studies have reported that [51] tumor cells can be ablated at temperatures higher than 48 °C in 5 min, due to the high temperature producing irreversible cell death of the tumor tissue. In the present study, our findings indicated that the high temperature generated by PEG-ReS_2_ nanosheets efficiently ablated the breast tumor after 10 min of PTT. The phototherapy outcome was preliminarily evaluated by investigating the H&E stained tumor slice images one day after treatment. As shown in Figure S1, the tumor tissue in Group 4 mice was seriously damaged and burned after exposure to the high temperature. Moreover, H&E staining of the Group 4 tumor slices revealed that the cell nuclei were broken and smaller than the normal morphology observed in the tumor slices from the other three groups (Figure 4c).

The tumor volume of tumor-bearing mice was measured every 2 days for 14 days following PTT treatment. As shown in Figure 4d, after PTT treatment the tumors in Group 4 mice rapidly shrank, and had disappeared by day 14. Compared with the other three groups, the Group 4 tumor size was significantly smaller (Figure 4e). H&E staining of the five major organs (hearts, liver, spleen, lungs, and kidney) indicated that there was no obvious organ damage, tumor metastasis, and visible inflammation (Figure S2). These observations also confirmed that PEG-ReS_2_ nanosheets had low in vivo toxicity and a satisfactory therapeutic outcome. It is worth noting that all the mice in the other three groups were euthanized on the 14th day, due to the large sizes of their tumors (Figure S3). The rapid growth of the tumors in the other three groups indicated both laser irradiation treatment alone or PEG-ReS_2_ nanosheet treatment alone did not efficiently inhibit tumor growth. Finally, we found that the PTT treatment involving the PEG-ReS_2_ nanosheets had no significant effect on the body weight of tumor-bearing mice (Figure 4f). Taken together, our findings confirm that PEG-ReS_2_ nanosheets have the potential to act as promising PTAs, which can be used to ablate tumors in vivo.

**Figure 5:**
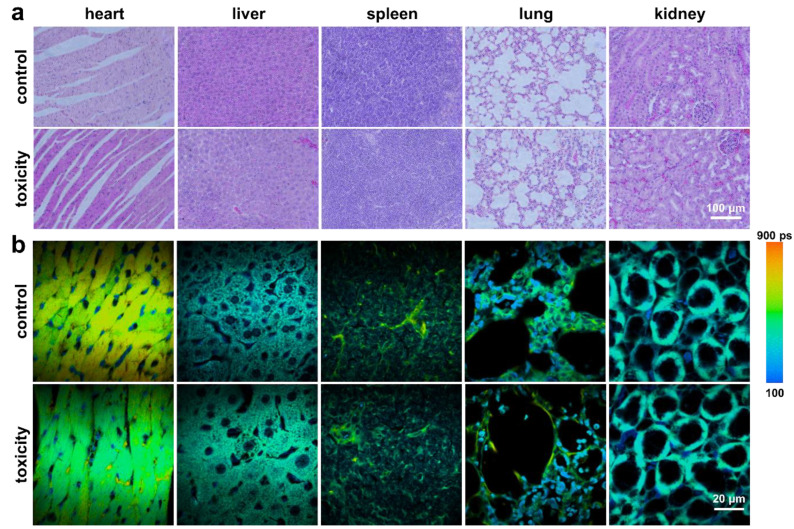
(**a**) Bright field images of tissue slices from five major organs including the heart, liver, spleen, lungs, and kidney after 2 months-post injection. (**b**) Fluorescence lifetime images of tissue slices from five major organs including the heart, liver, spleen, lungs, and kidney after 2 months-post injection. Note that the pseudo-color scale bar in Figure 5b represents the lifetime distribution interval from 100 to 900 ps.

Next, we confirmed low in vivo toxicity of PEG-ReS_2_ nanosheets through the intravenous injection method. Two healthy BALB/c mice were injected with 100 μL PEG-ReS_2_ nanosheets (6 mg/mL in water) via the tail vein. Two months later, the PEG-ReS_2_ nanosheets were found to accumulate in the major organs via blood circulation. However, H&E staining revealed no significant organ damage or visible inflammation due to the accumulation of PEG-ReS_2_ nanosheets (Figure 5a). Moreover, changes in the tissue and cells’ microenvironment were examined by extracting fluorescence lifetime changes by FLIM [52] (Figure 5b). No obvious differences in the lifetime distributions of the control group and toxicity group were observed, further highlighting the low In vivo toxicity of PEG-ReS_2_ nanosheets.

**Figure 6:**
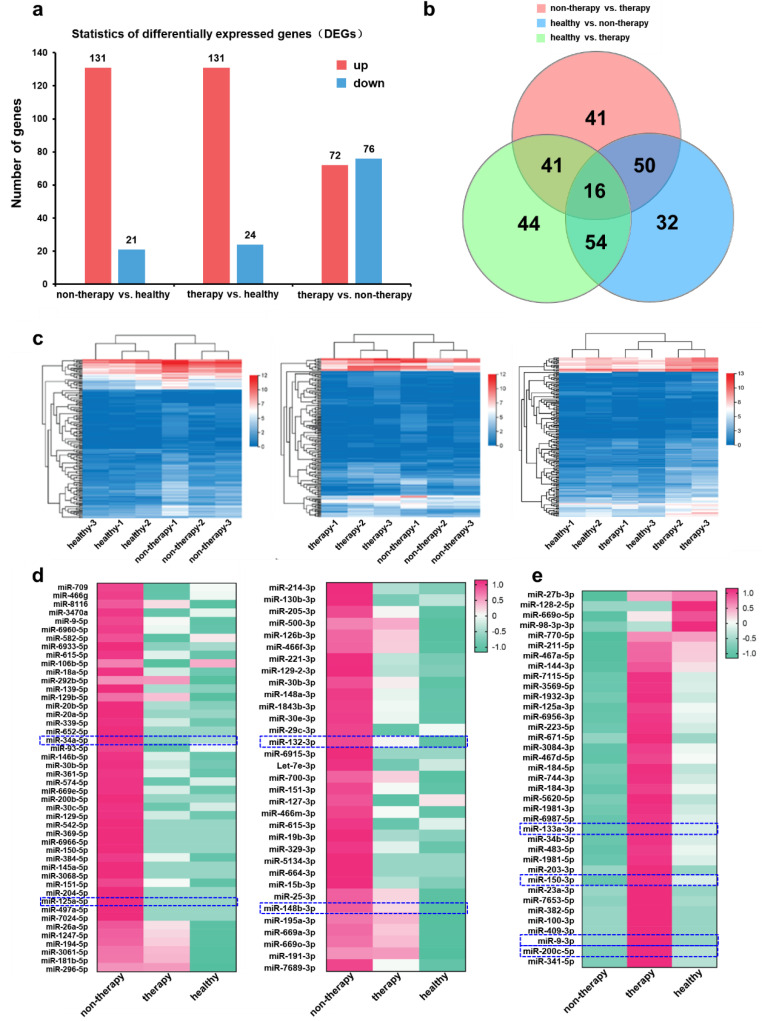
miRNA expression analysis. (**a**) Statistical analysis of differentially expressed genes (DEGs) from non-therapy vs. healthy group, therapy vs. healthy group, and therapy vs. non-therapy group, respectively. (**b**) Plot showing the differentially expressed genes from two different groups in the non-therapy group, therapy group, and healthy group. (**c**) DEGs cluster heatmaps showing healthy group vs. non-therapy group, therapy group vs. non-therapy group, and healthy group vs. therapy group, respectively. (**d**) The down-regulated miRNAs caused by PEG-ReS_2_-based PTT in non-therapy group, therapy group, and healthy group, respectively. (**e**) The up-regulated miRNAs caused by PEG-ReS_2_-based PTT in non-therapy group, therapy group, and healthy group, respectively.

miRNA expression analysis was performed to further evaluate the therapeutic outcome of PEG-ReS_2_ nanosheets. Changes in miRNA expression levels usually correspond to tumor generation or shrinkage. Thus, miRNA expression levels are important indicators for screening cancer treatments. As shown in Figure 6a, significant changes in the expression levels of a total of 278 miRNAs were observed when comparing the non-therapy (PBS treatment alone), therapy (ReS_2_ + NIR), and healthy groups. Significant changes in miRNA expression levels were observed during BC tumor development including up-regulation of 131 miRNAs and down-regulation of 21 miRNAs in mice suffering from BC (non-therapy group) compared to healthy mice. In addition, 131 up-regulated miRNAs and 24 down-regulated miRNAs were found in the therapy group compared to healthy mice. Finally, 72 miRNAs were up-regulated and 76 miRNAs were down-regulated in the PEG-ReS_2_-based PTT therapy group compared to the non-therapy group. As shown in Figure 6b, almost half of the DEGs can be found in at least two groups. Furthermore, approximately 16 DEGs were simultaneously found in all three study groups, indicating that these 16 DEGs may be involved in the PTT process. The DEGs cluster heatmaps shown in Figure 6c indicate that each BALB/c mouse in the healthy group could not be clustered with the BALB/c mice in the non-therapy group. In addition, there was no obvious correlation between the BALB/c mice in the therapy group and the BALB/c mice in the non-therapy group. These heatmaps revealed that the miRNA expression levels in the non-therapy group were distinct from both the healthy and therapy groups. Interestingly, the right heatmap indicated that the miRNA expression in the therapy group was similar to that observed in the healthy group, especially for sample therapy-1. Thus, our data suggest that some miRNA expression patterns in tumor-bearing mice return to normal levels after PEG-ReS_2_-based PTT. 

In order to evaluate the similarity of the whole miRNA expression levels among non-therapy, therapy, and healthy groups, Kyoto Encyclopedia of Genes and Genomes (KEGG) pathway analysis were performed, providing data as shown in Figure 6d,e. Compared to the non-therapy group, 76 miRNAs were down-regulated in the therapy group, while 131 miRNAs were down-regulated in the healthy group as shown in Figure 6a. Among the union of these miRNAs, 79 miRNAs were further subtracted by using KEGG analysis, which were shown as in Figure 6d. Similarly, compared to the non-therapy group, 72 genes up-regulated miRNAs in the therapy group and 21 genes up-regulated miRNAs in healthy group were also analyzed, generating Figure 6e. The whole miRNA expression levels of Figure 6d revealed a high similarity between the non-therapy group and the healthy group, which indicates a recovery of the tumor-induced expression after PEG-ReS2-based PTT in mouse models. Typical miRNAs, which are highly correlated with cancer based on the KEGG pathway analysis, were identified based on the human miRNA database (Figure S4). KEGG enrichment analysis revealed that many of these down-regulation miRNAs including miR-125a-5p [53,54], miR-34a-5p [55], miR-132-3p [56], and miR-148b-3p, were BC biomarkers highly correlated with PEG-ReS2-based PTT. In addition, we found 37 up-regulation miRNAs (Figure 6e and Figure S5), including miR-133a-3p [57,58], miR-200c-5p, miR-9-3p, and miR-150-3p, which were also highly correlated with PEG-ReS_2_-based PTT. These findings indicated that both up-regulation and down-regulation miRNAs involved in BC were important biomarkers for assessing the therapeutic outcomes of a PTT treatment based on PEG-ReS_2_ nanosheets. Thus, miRNA expression level analysis can provide a new insight into evaluating the therapeutic outcomes of low dimensional nanomaterials-based cancer therapy.

## 4. Conclusions

In this study, we successfully constructed promising colloidal PEG-ReS_2_ nanosheets, which can be used for In vivo BC therapy. The PEG-ReS_2_ nanosheets had a strong absorbance band at 834 nm, indicating that they possessed strong NIR light absorption capability. In addition, the PEG-ReS_2_ nanosheets had good solubility and low toxicity. Moreover, the PEG-ReS_2_ nanosheets possessed a high PTCE of 42% after irradiation with an 808 nm laser. More importantly, the PEG-ReS_2_ nanosheets were found to promote cell death in vitro in 4T1 BC cell lines and ablate in vivo tumors through remarkable photothermal effects. In addition to the examination of H&E stained tissue slices, the miRNA expression analysis method was employed to evaluate the therapeutic outcomes of PEG-ReS_2_ nanosheets. It can be expected that the PEG-ReS_2_ nanosheets may be a promising PTA for future BC therapy. The miRNA expression level analysis provided a novel insight into evaluating the therapeutic outcomes of PEG-ReS_2_ nanosheets through molecular biology mechanisms. Both down-regulated miRNAs (miR-125a-5p, miR-34a-5p, miR-132-3p, and miR-148b-3p) and up-regulated miRNAs (miR-133a-3p, miR-200c-5p, miR-9-3p, and miR-150-3p) were important biomarkers for confirming good therapeutic outcomes of PEG-ReS_2_ nanosheets. The proposed PEG-ReS_2_ nanosheets showed great promise in cancer therapy, and miRNA expression level analysis could potentially provide a whole assessment for the therapeutic effect of low-dimensional nanomaterials-based cancer therapy in the mouse model as well as a comparison with the miRNA profiling data from clinical breast cancer cases.

## Data Availability

The data presented in this study are available upon request from the corresponding author.

## Supplementary Materials

The following supporting information can be downloaded at: https://www.mdpi.com/article/10.3390/nano12111937/s1. Figure S1: The obtained tumors from four study groups on the second day after PTT. Figure S2: Optical images of H&E stained sections obtained from five vital organs. The images were captured by a 20× objective. Figure S3: The tumors from mice in non-treated group the 14-th day after PTT. Figure S4: The KEGG pathway enrichment analysis on the down-regulated miRNAs. Figure S5: The KEGG pathway enrichment analysis on the up-regulated miRNAs.

## Abbreviations

PTT, photothermal therapy; PEG, poly-ethylene glycol; NIR, near-infrared; PTCE, photothermal conversion efficiency; PTAs, photothermal transduction agents; miRNAs, microRNAs; NPs, nanoparticles; PDT, photodynamic therapy; 2D, two-dimensional; TMDs, transition metal dichalcogenides; CT, computerized tomography; BC, breast cancer; AM, Acetoxymethyl ester; PI, propidium iodide; NMP, N-Methyl-2-pyrrolidinone; TEM, transmission electron microscopy; CO_2_, carbon dioxide; OD, optical density; H&E, hematoxylin and eosin; PBS, phosphatic buffer solution; TPM, transcripts per kilobase million; DEG, differentially expressed gene; vdW, Van der Waals; FLIM, fluorescence lifetime imaging microscopy; KEGG, Kyoto Encyclopedia of Genes and Genomes.
