# Supplementary material for: Promising Colloidal Rhenium Disulfide Nanosheets: Preparation and Applications for In Vivo Breast Cancer Therapy
Source: Nanomaterials (Basel). 2022 Jun 6;12(11):1937. doi: 10.3390/nano12111937 (PMC9182237; doi:10.3390/nano12111937)
Supplement: Supplementary file 1 [file nanomaterials-12-01937-s001.zip › nanomaterials-1717785-supplementary.pdf]

nanomaterials

Supplementary Materials

Promising Colloidal Rhenium Disulfide Nanosheets: Preparation and Applications for In Vivo Breast Cancer Therapy

Yiwan Song [1], Yufeng Yuan [1,2,*], Xiao Peng [1,*], Zheng Peng [1], Hao Liu [1], Yingxin Zhou [1], Xiaoying Zhang [3], Feifan Zhou [1], Jun Song [1] and Junle Qu [1]

[1] College of Physics and Optoelectronic Engineering, Shenzhen Key Laboratory of Photonics and Biophotonics, Key Laboratory of Optoelectronic Devices and Systems of Ministry of Education and Guangdong Province, Shenzhen University, Shenzhen 518060, China; yiwansong_syw@163.com (Y.S.); zheng_peng1995@163.com (Z.P.); 20222022@163.com (H.L.); yls772@163.com (Y.Z.); ffzhou@szu.edu.cn (F.Z.); songjun@szu.edu.cn (J.S.); jlqu@szu.edu.cn (J.Q.)
[2] School of Electronic Engineering and Intelligentization, Dongguan University of Technology, Dongguan 523808, China
[3] Department of Pharmacology, College of Pharmacy, Shenzhen Technology University, Shenzhen 518118, China; zhangxiaoying@sztu.edu.cn
* Correspondence: yufengyuan@dgut.edu.cn (Y.Y.); pengxiao_px@szu.edu.cn (X.P.)

1. Supplementary Equation

$$\theta = \frac{\Delta T_i}{\Delta T} \tag{S1}$$

$$-\ln(\theta) = \frac{t}{\tau_s} \tag{S2}$$

$$\tau_s = \frac{m_D c_D}{hS} \tag{S3}$$

where, the parameter θ is defined by the ratio between ΔT_i and ΔT_{max}. Here, ΔT_i means the difference between the temperature at a different record time and final temperature. ΔT_{max} represents the maximum difference value of the temperature in cooling process. The t in equation (2) means the recorded time. Then, the slope of fitting curve between t and -ln(θ) is the value of τ_s. As shown in equation (3), the m_D stands for the mass of PEG-ReS_2 aqueous solution, and c_D represents the specific heat capacity of water. Eventually, the hS could be determined by the experiment data.

2. Supplementary Figures

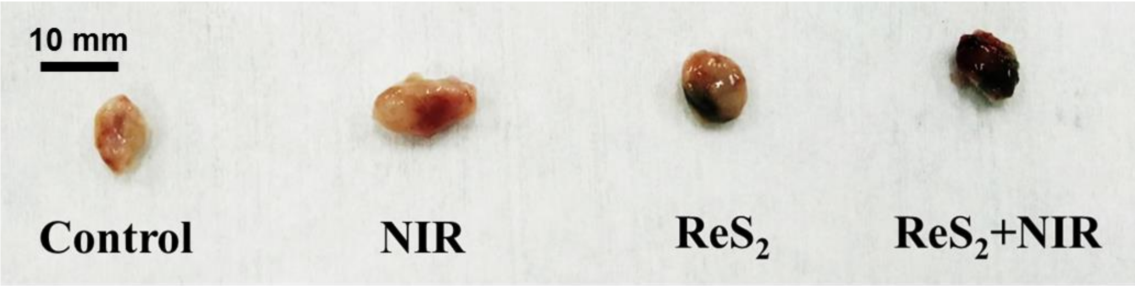

Figure S1. The obtained tumors from four study groups on the second day after PTT.

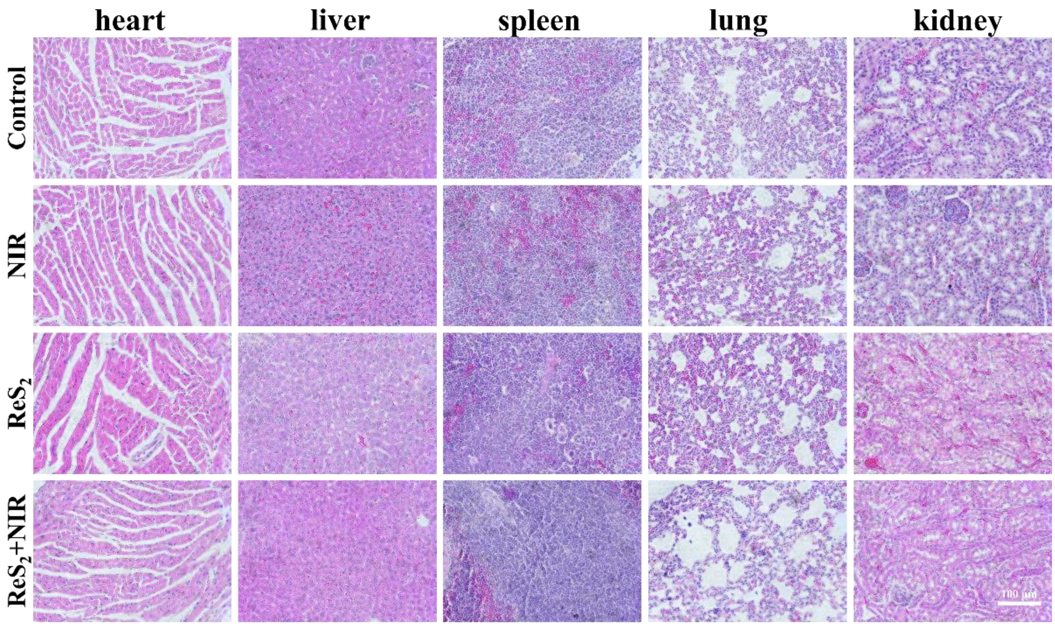

Figure S2. Optical images of H&E stained sections obtained from five vital organs. The images were captured by a 20× objective.

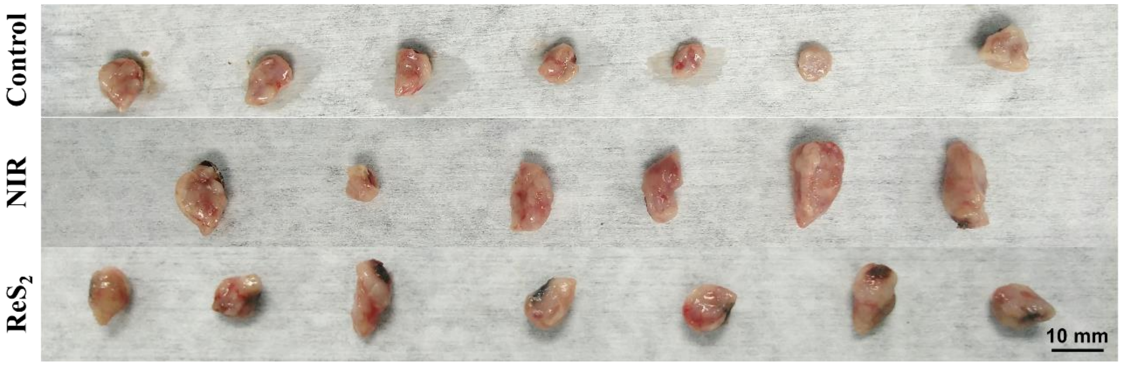

Figure S3. The tumors from mice in non-treated group the 14th day after PTT.

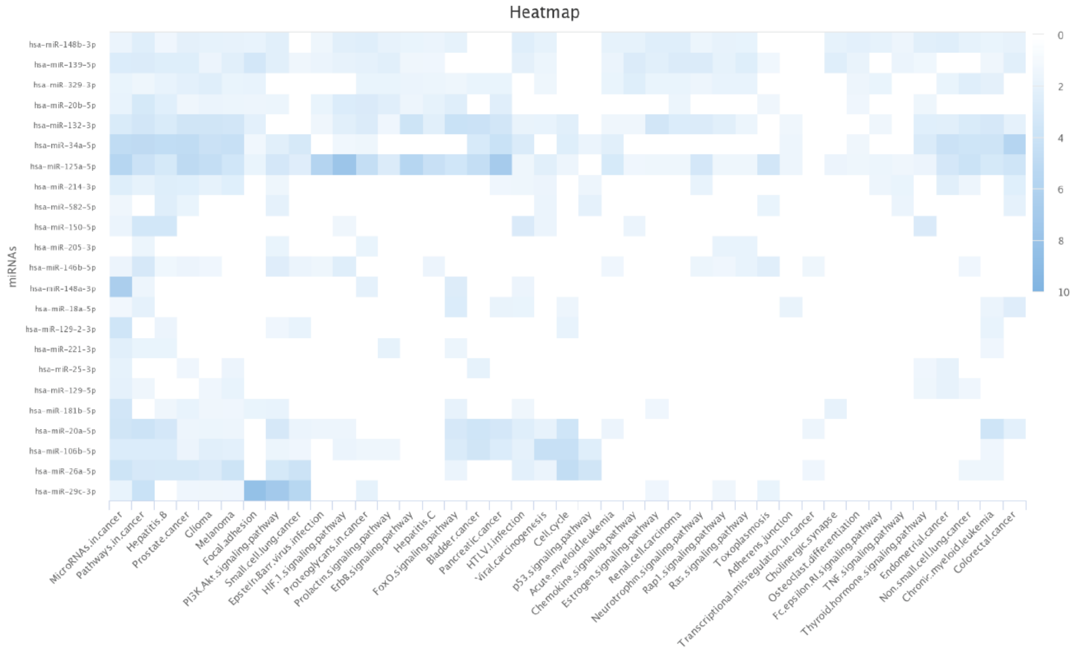

Figure S4. The KEGG pathway enrichment analysis on the down-regulated miRNAs.

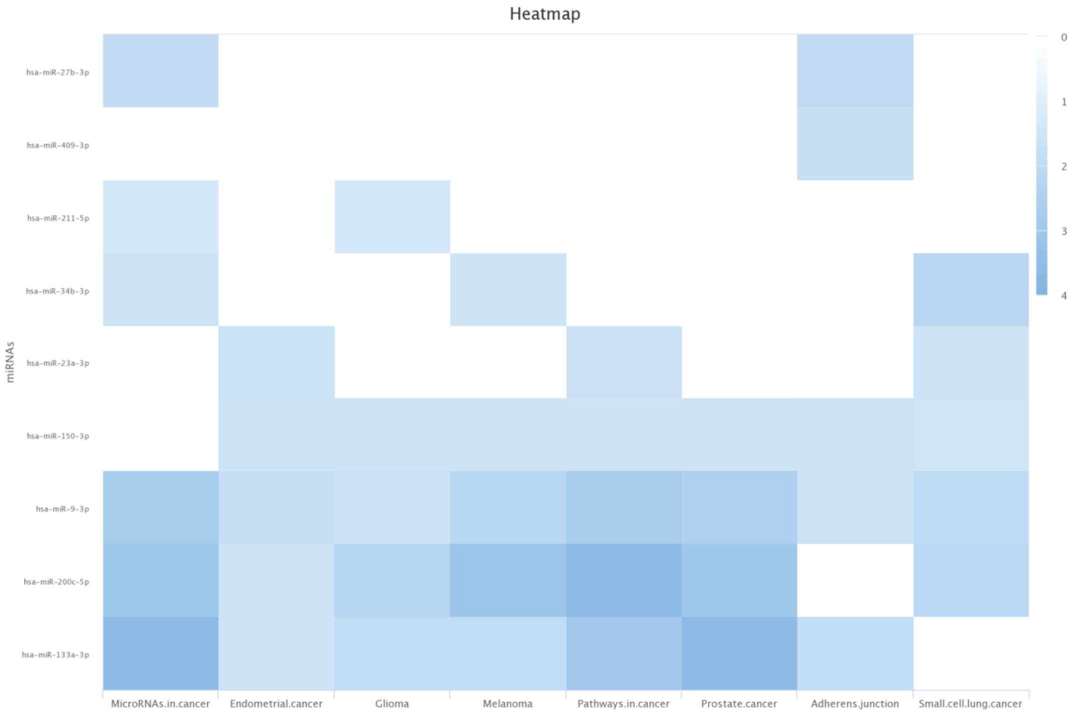

Figure S5. The KEGG pathway enrichment analysis on the up-regulated miRNAs.
